# Recent Advances in Seismocardiography

**DOI:** 10.3390/vibration2010005

**Published:** 2019-01-14

**Authors:** Amirtahà Taebi, Brian E. Solar, Andrew J. Bomar, Richard H. Sandler, Hansen A. Mansy

**Affiliations:** 1 Department of Biomedical Engineering, University of California Davis, One Shields Ave, Davis, CA 95616, USA; 2 Biomedical Acoustics Research Laboratory, University of Central Florida, 4000 Central Florida Blvd, Orlando, FL 32816, USA; 3 College of Medicine, University of Central Florida, 6850 Lake Nona Blvd, Orlando, FL 32827, USA

**Keywords:** seismocardiography, heart-induced vibrations, cardiovascular disease, signal processing, signal segmentation, noise removal, feature extraction, machine learning

## Abstract

Cardiovascular disease is a major cause of death worldwide. New diagnostic tools are needed to provide early detection and intervention to reduce mortality and increase both the duration and quality of life for patients with heart disease. Seismocardiography (SCG) is a technique for noninvasive evaluation of cardiac activity. However, the complexity of SCG signals introduced challenges in SCG studies. Renewed interest in investigating the utility of SCG accelerated in recent years and benefited from new advances in low-cost lightweight sensors, and signal processing and machine learning methods. Recent studies demonstrated the potential clinical utility of SCG signals for the detection and monitoring of certain cardiovascular conditions. While some studies focused on investigating the genesis of SCG signals and their clinical applications, others focused on developing proper signal processing algorithms for noise reduction, and SCG signal feature extraction and classification. This paper reviews the recent advances in the field of SCG.

## 1. Introduction

Cardiovascular disease results in one death every 40 s in the United States [1]. Improved diagnostic, surveillance, and intervention methods would help reduce mortality [2,3] and extend lives. Heart disease may be detected using many non-invasive methods including manual auscultation of heart sounds, which is a common component of physical examinations and known to provide useful diagnostic information. However, simple auscultation of heart sounds is of limited utility. Detection and processing of very-low-frequency heart sounds (“infrasounds”) below the limit of human ear detection may extend the diagnostic power of auscultation. New studies of cardiac-generated sounds using computational fluid dynamics [4,5] and advanced signal processing methods suggested increased potential to provide quantitative information that may be helpful for patient monitoring and diagnosis. Seismocardiography (SCG) is a noninvasive technique that measures cardiac-induced mechanical vibrations at the chest surface including those below the human hearing threshold. The reader is referred to previous SCG reviews [6,7] describing earlier studies, while this paper reviews more recent SCG studies including advances in instrumentation and signal processing that hold the promises of increased clinical utility. Since previous reviews of SCG [6,7] described early SCG studies, this article is more focused on the developments in the field during the last few years. Between September 2017 and March 2018, we conducted a search of the scientific journals and conferences using MEDLINE, as well as the Google Scholar search engine, for the following expressions: “SCG”, “seismocardiography”, and “seismocardiogram”. Reviewing the reference section of the initial results led to additional articles. More resources were added during the manuscript preparation and revision. The final sampling period includes studies that were published after [7] and before November 2018.

### 1.1. Definition of SCG Signals

The measurements of heart-induced motion, including displacement, velocity, and acceleration, were performed as early as the turn of the 20th century [8]. These approaches can be categorized into two classes [6,7]: (a) the measurement of whole-body recoil forces in response to cardiac ejection, usually termed ballistocardiography (BCG); and (b) the local chest surface measurement of cardiac-induced vibrations, typically referred to as SCG [9–13]. These vibrations are usually measured in the form of acceleration (m/s^2^). This article focuses on the latter.

### 1.2. Physiological Sources of SCG Signals

SCG signals are believed to be caused by cardiac mechanical processes including cardiac muscle contraction, cardiac valve movement, blood flow turbulence, and momentum changes. The characteristics of these signals are likely to contain useful information that correlate with cardiovascular physiologic [14] and pathologic processes [15]. Such information may powerfully complement methods of detecting heart electrical activity (such as electrocardiography), serologic testing, and imaging modalities (e.g., echocardiography, cardiac magnetic resonance imaging (MRI), and catheterization).

Early BCG studies [8] suggested that heart-induced motion may be used to estimate changes in cardiac output, and reported certain signal patterns in patients with myocardial infarction [16]. These signal patterns were also found to correlate with the strength of myocardial contractions [17,18] and contain detectable waveform changes during heart disease resolution [19]. After introducing SCG in early 1990s [13], recent studies suggest possible SCG utility for monitoring left-ventricle function, coronary blood flow during balloon angioplasty [9,10,20], heart and breathing rates [21–26], and ventricular filling, cardiac valve closure, and ventricular ejection preceding the onset of ischemic symptoms [10].

While the relationship between SCG waves and cardiac activity is not fully understood, several studies investigated this relationship. For example, SCG was reported to contain a low-frequency wave during atrial systole, a high-amplitude wave during ventricular systole, another wave during early ventricular filling, and some relatively high-frequency waves at the time of the first and second heart sounds [13]. Simultaneous recording of SCG and electrocardiogram (ECG) indicated that the peaks and valleys of the SCG correspond to known physiological events including mitral valve opening (MO) and closure (MC), isovolumetric contraction, ejection, aortic valve opening (AO) and closure (AC), and cardiac filling [7,27]. The utility of SCG in estimating cardiac intervals such as electromechanical systolic pre-ejection period (PEP) and left-ventricular ejection time (LVET) was also shown [28]. Multi-channel partially simultaneous SCG, ECG, and sonographic measurements were used to identify the feature points in a cardiac cycle corresponding to the four common valvular auscultation locations. Using these measurements, new feature points (including left-ventricular lateral wall contraction peak velocity, septal wall contraction peak velocity, trans-aortic valve peak flow, transpulmonary peak flow, trans-mitral ventricular relaxation peak flow, and trans-mitral atrial contraction peak flow) were reported.

**Figure 1.**
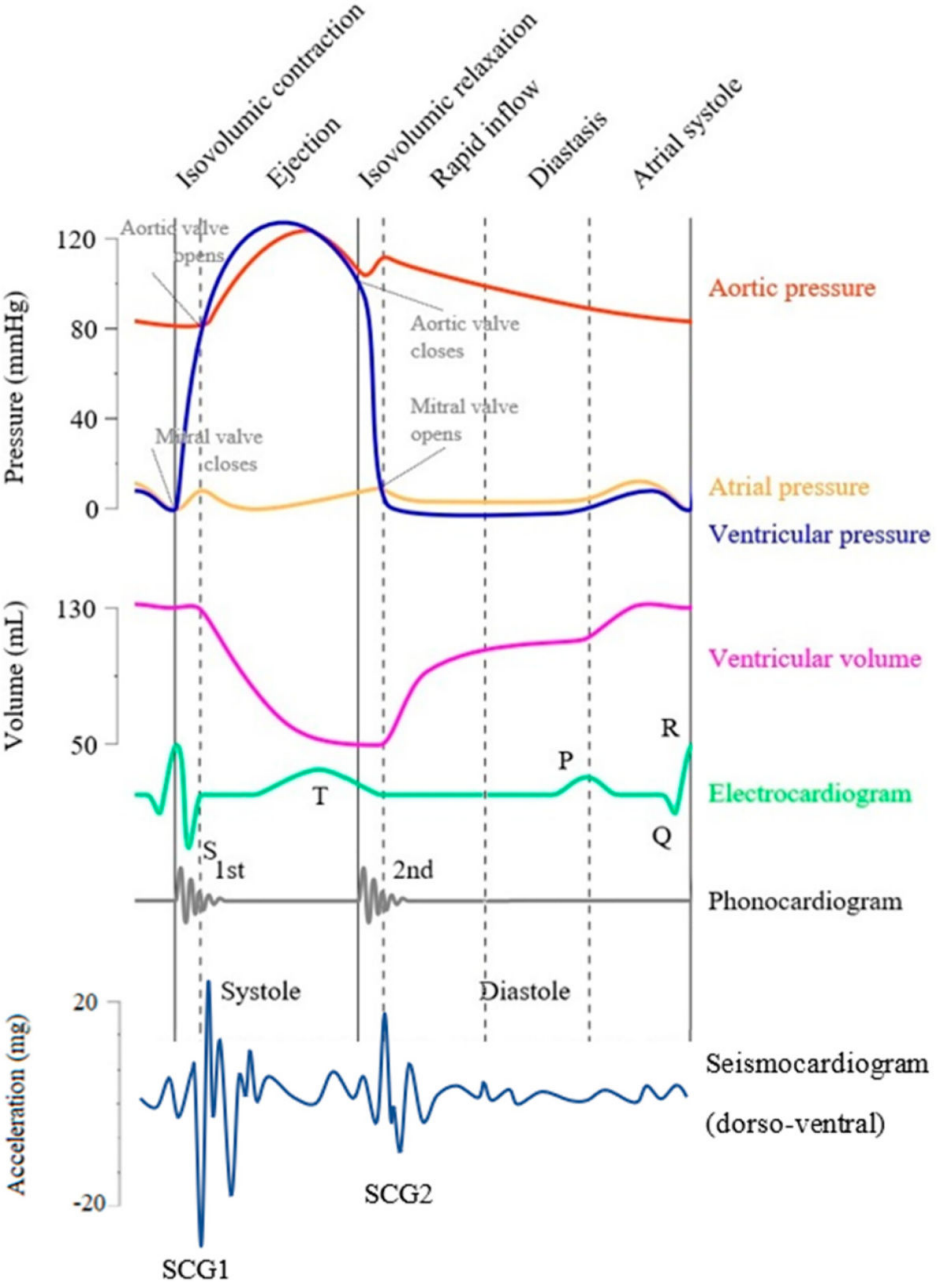
Modified Wiggers diagram. A sample axial seismocardiography (SCG) signal (acceleration in the dorso-ventral direction) is shown alongside other cardiovascular signals such as the aortic pressure, atrial pressure, ventricular volume, electrocardiograme and phonocardiogram. The mitral valve closure (MC) and opening (MO), and aortic valve closure (AC) and opening (AO) are labeled based on the pressure signals.

Table 1 lists all the SCG feature points and cardiac time intervals (CTIs) that were reported in the literature, while Figure 1 shows a modified Wiggers diagram [29] where a sample SCG signal (in the dorso-ventral direction) is plotted along with aortic blood pressure, ventricular volume, and the electrocardiogram.

During the cardiac cycle of healthy individuals, the apex and base rotate in opposite directions, which results in a twisting motion of the left ventricle [35] known to be affected by different factors such as aging and diastolic dysfunction. Investigating the rotational vibration induced by this heart twisting motion might provide complementary information to the current SCG analysis of uni- and triaxial accelerations. In recent studies [31,36,37], a three-axis micro electromechanical systems (MEMS) gyroscope and a three-axis accelerometer were used simultaneously to measure the rotational and axial components of chest vibrations. The potential utility of the combined analysis of axial and rotational heart-induced vibrations were suggested for the ECG-independent identification of systolic points (such as AO and AC) and cardiac time intervals (such as LVET and PEP) [38].

In summary, despite many studies conducted about SCG genesis, the relationship between SCG waves and cardiac activity is not yet fully understood. This is possibly because of the waveform variations in different studies and lack of understanding of the exact SCG waves sources. Thus, there is still a need for widely accepted universal labeling (i.e., valid for all/majority of patients) analogous to PQRST labeling in ECG.

### 1.3. Measurement Methods

New advances and availability of lightweight low-noise accelerometers improved the quality of recorded SCG signals. Different methods were used for SCG measurement in the recent studies, including the following:

- Uniaxial/triaxial piezoelectric accelerometers [39–42];
- Uniaxial/triaxial MEMS accelerometers [36,43–45];
- Smartphone accelerometers and gyroscopes [46–48];
- Triaxial gyroscopes [31,36,37,45];
- Laser Doppler vibrometers [42,49];
- Microwave Doppler radars [50–52];
- Airborne ultrasound surface motion camera (AUSMC) [53].

Depending on the sensors that are used, SCG signals might consist of one or more axial and rotational components. For example, a uniaxial accelerometer can be used to measure SCG component in the dorso-ventral direction. However, combination of a triaxial accelerometer and triaxial gyroscope can provide information about axial and rotational heart-induced motion in three different directions. This review focuses on the dorso-ventral component of the SCG signal, unless otherwise stated.

**Figure 2.**
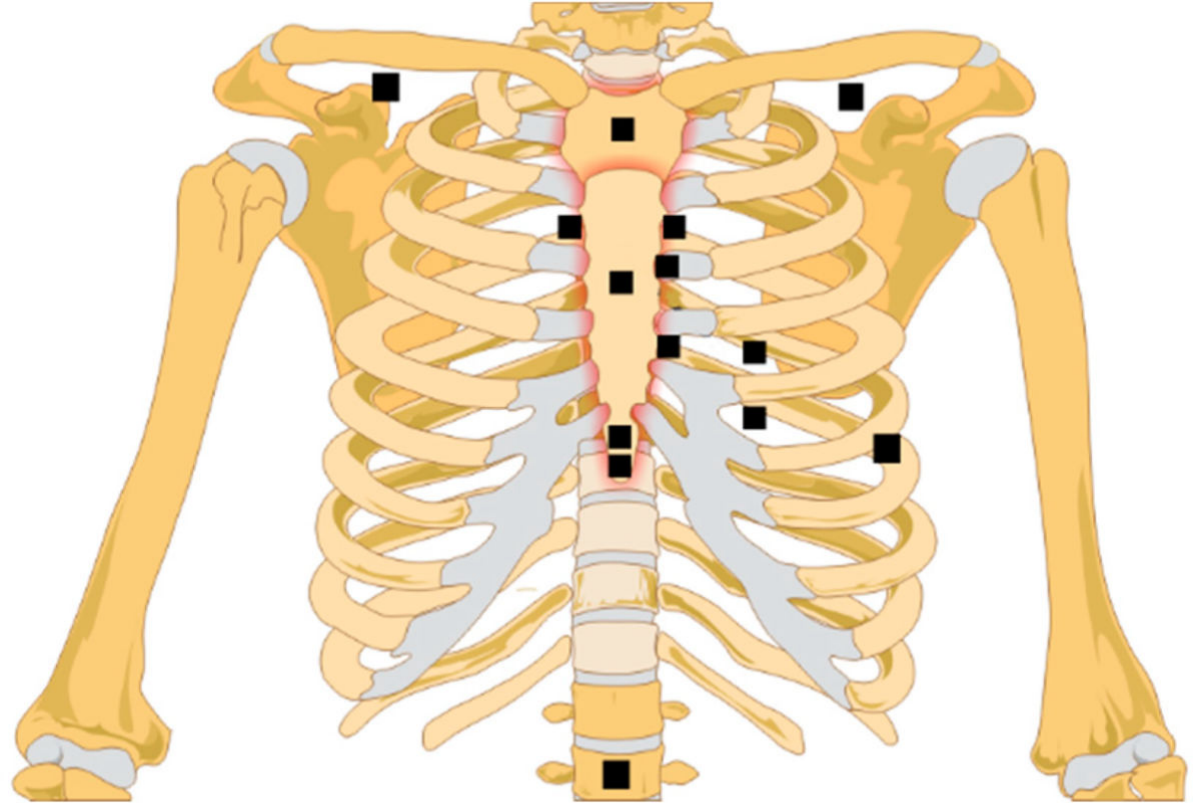
Sensor location distribution in recent SCG studies.

Sensors are most commonly placed on (or directed to) the sternum or its left lower border. However, in some studies, other locations were used for SCG signal acquisition, including over the heart apex (lateral left lower chest) and the “aortic valve listening area” at the right upper sternal boarder [34,43,44]. Information about sensor type, model, and placement location in recent studies is summarized in Table 2 and Figure 2.

In some applications, such as burn patients, highly infectious patients, and premature babies, attaching adhesive ECG electrodes or SCG sensors would not be feasible. Therefore, development of efficient contactless SCG detection techniques are under investigation. These techniques include laser Doppler vibrometry (LDV), microwave Doppler radar, and airborne ultrasound imaging [22,53,84]. A non-contact SCG measurement might also reduce skin coupling; artefacts that may tie present in the SCG signals acquired by the contact sensors attached to the skin.

The LDV approach compares the frequency shift between the outgoing and reflected laser beams and determines the corresponding vibration velocity of the surface that reflected the beam [85]. Considerations when using LDV for SCG measurements include the following: (1) the chest surface needs to be reasonably reflective for accurate LDV measurements, (2) the laser beam should be perpendicular to chest surface, and (3) chest movement due to respiration needs to be accounted for, since breathing causes the point of measurement to be displaced in the chest: plane. One solution to this issue is to develop an algorithm that can automatically have the beam follow a measurement point on the chest surface. Other LDV limitations include their cost and size.

Microwave Doppler radar is another non-contact method that can be used for SCG measurements. When recording the SCG signal using microwave Doppler radars, the SCG will exhibit in the phase variation of the microwave signal· SCG signals can then be extracted from this phase variation. Like LDV sensors, Doppler radar approaches have the benefit of contactless signal acquisition, but suffer from the reflection of background microwave signals (called radar clutter) lowering the signal-to-noise ratio [51].

The utility of ultrasound imaging in estimating non-contact two-dimensional (2D) SCG maps of the body surface was also investigated [53]. In addition to the advantages of other contactless measurement methods, this technique can collect SCG data from multiple locations through different channels resulting in a potentially higher reliability. However, this method requires a planar measurement surface that is parallel to the emission panel.

Piezoelectric and MEMS sensors are smaller and lighter than contactless sensors. Therefore, these sensors might be used in clinical settings for everyday and continuous screening subjects suspected of different cardiovascular diseases.

### 1.4. Parameters Affecting SCG Waveform

A main challenge in SCG studies is that SCG signal morphology appears to very significantly, not only by cardiovascular pathology, Cut also normal inter-subject variation. These changes are affected by several factors including respiratory cycle phases, gender, age, sensor chest location, health conditions, cardiac contractility, heart rhythm, and postural positions [23,25,86,87]. While these changes can lead to undesirable SCG variability, deeper understandings of these processes will enhance our understanding oi SCG signal s, help aggregate SCG cycles into groupings with similar SCG events to reduce SCG signal variability and noise, and hopefully lead to more accurate definition of SCG features for diagnoses and monitoring.

A few studies addressed SCG variability, e.g., the consistent effects of respiration [11,32]. One recent study [66] reported that the SCG morphology appeared to mainly depend on the lung volume (and, hence, possibly the intrathoracic pressure), rather than dependence on negative or positive airflow (i.e., inspiration or expiration). This SCG morphology variation can also be used to automatically identify the lung volume states and respiratory phases by employing machine learning [56]. Another study used support vector machines to classify the SCG cycles occurring during the high and low lung volumes [40]. Successful grouping of SCG cycles into two groups, where SCG events in each group are more similar to each other and dissimilar to the events in the alternate group, would improve the signal-to-noise ratio in calculating the SCG ensemble average. This would result in more accurate estimation of diagnostic information from the SCG ensemble average.

Subject motion and postural position were also shown to cause changes in SCG signals [7,75,88]. In an ongoing study, the effect of posture on the SCG signals was investigated for patients with heart failure (HF) [75]. The SCG signals were measured using a wearable unit in supine and seated positions. The SCG power spectral density (PSD) was estimated using Welch’s periodogram, and the means of PSD values were calculated in the 0–20-Hz band. The results showed that SCG signals contained high energy in bands greater than 8 Hz in the supine and seated postures. Identification of chest orientation (supine, 45°, or vertical), and, therefore, grouping of SCG signals according to chest orientation is possible with the use of certain triaxial accelerometers [88]. However, chest orientation measurement is not sufficient to account for all postural changes, as shown by the reported SCG differences between sitting upright and standing, which have the same chest orientation. Movement of the patient also produces a change in the SCG signal. The ability to filter noise originating from speaking, walking, and indistinct motions associated with workplace tasks was demonstrated [43]. Understanding the effects of posture and movement on the SCG waveform is a useful step toward continuous collection of SCG signals from a patient from a wearable sensor. However, this may not be needed if intermittent testing in a more controlled environment is performed at a fixed subject position.

Exercise and the following period of recovery was also demonstrated to produce changes in SCG signals. Not surprisingly, exercise is associated with an increase in the overall amplitude of the SCG signal, measured as the root-mean-square (RMS) power [89,90]. This increase in signal amplitude was shown to correlate with increased cardiac output observed during exercise [89]. This cardiac output increase is a result of increased heart rate and stroke volume. Exercise also produces changes in the left-ventricular ejection time (LVET) and the pre-ejection period (PEP) [88,90]. As exercise increases the heart rate (also seen as the R-R interval decrease in ECG), it generally causes a decrease in other measured time intervals such as LVET and PEP. LVET correlates with both heart rate and contractility and, hence, decreases with exercise. PEP is less affected by heart rate but does decrease during exercise due to the increased contractility (inotropy). These changes in LVET and PEP were detectable by SCG [88,91], and exercise-induced decrease in PEP was found to shift the SCG signal power spectrum toward higher frequencies [78].

Digestive state and mood may affect cardiac function through similar physiological mechanisms, thereby possibly affecting SCG signal morphology. Systematic investigations of these effects are lacking, and future studies are needed to determine the magnitude and nature of these effects on the SCG signal.

The sensitivity of the SCG signal to sensor location is well known and, therefore, needs to be taken into account when comparing results from different studies. Historically, investigators placed accelerometers at different anatomical locations, including the clavicle, the sternum, and various intercostal spaces [34,88,89]. A recent study [34] investigated the differences in SCG signals morphology at the common auscultation sites of the four heart valves (aortic, pulmonary, tricuspid, and mitral), and found significant differences in SCG morphology. That study also concluded, with the aid of sonographic measurements, that more feature points can be defined from multi-point SCG measurements.

Due to the sensitivity of SCG signals to the measurement location, unexperienced users might not be able to repeatably record the SCG at ideal locations. Hence, the SCG-based estimations of cardiac activity might change significantly due to sensor location errors. This can, in turn, result in inaccurate interpretations [41]. To overcome this issue, Ashouri and Inan [41] proposed a method to automatically detect when the sensor is not placed in a desired location by comparing the regression parameters from the acquired SCG and an SCG measured from a reference position.

**Figure 3.**
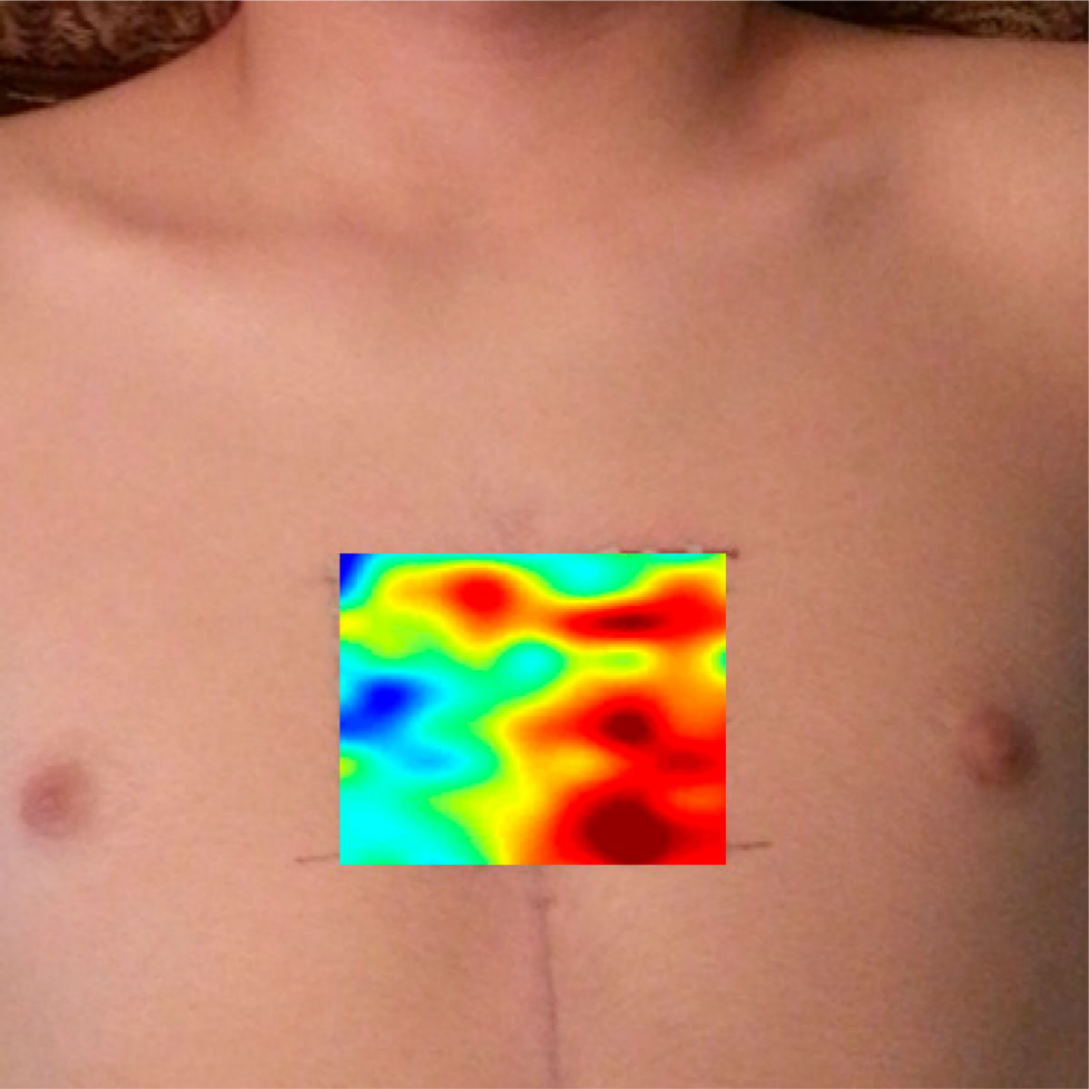
Map of root-mean-square (RMS) amplitude of SCG waves at the chest surface using scanning laser vibrometry. There were local amplitude maxima that coincided with the aortic, pulmonary, tricuspid, and mitral auscultation areas. These data suggest that sensor location and size need to be chosen with care and that the effects of sensor misplacement need to be quantified.

High-spatial-resolution measurement of the SCG signal was carried out in a pilot study Here, a laser vibrometer was used to perform non-contact uniaxial SCG measurements in the dorso-ventral direction. The laser beam was <2 mm^2^ and the resulting SCG amplitude distribution is shown in Figure 3. These data suggest that, when the sensor location changed by 1 cm, the SCG amplitude can vary by >30%. An accelerometer with larger contact crea (3.5 cm^2^) was also used and it was found that, for a sensor location change of 1 cm, the SCG amplitude changed by about 5%. Due to this smaller change, the larger contact area may be beneficial in reducing SCG dependence on sensor placement. The effects of sensor contact area and SCG spatial distributions (including axial or rotational signals) need further investigations.

## 2. Signal Processing

**Figure 4.**
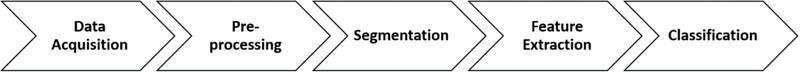
SCG signal processing steps.

SCG signal processing usually consists of several steps including preprocessing (e.g., down-sampling and denoising), signal segmentation, feature extraction, and classification (Figure 4). There were several recent studies that focused on noise removal, segmentation, and feature extraction of SCG signals. These studies are reviewed in this section.

### 2.1. Noise Reduction

While SCG signals can contain useful diagnostic information, they are often contaminated by noise from different sources including sensor mechano-electronics, motion artefacts, and environmental vibrations. This signal contamination might result in errors in calculating SCG features and eventually inaccurate signal classification, especially if automated SCG processing is performed (i.e., without human supervision). For example, a recent study [92] showed that, when determining the instantaneous frequency of SCG signals using different time-frequency distributions, estimation accuracy differed significantly with the signal-to-noise ratio. These results indicated that some time-frequency distributions performed poorly in noisy conditions and would lead to inaccurate time-frequency features. It was then concluded that feature extraction methods might fail or, at a minimum, perform inaccurately for low signal-to-noise ratio conditions.

Most research groups applied conventional band-pass filters to remove baseline wandering, body movements, and breathing artefacts from SCG signals [26,36,38,41,45,46,55,58–63,67,71,75,76,78–80,82,93]. A few studies utilized or proposed more advanced noise removal techniques [64,76,88,94–96]. A recent study [94] proposed a filtering algorithm based on the ensemble empirical mode decomposition (EEMD) to remove white Gaussian noise from SCG signals. This algorithm provided a higher signal-to-noise ratio than other filters such as Wiener filters. In a different study [76], a filtering algorithm based on empirical mode decomposition (EMD) was suggested to filter the SCG signals recorded during walking from a wearable device. This EMD-based denoising approach appeared to result in better estimations of PEP during walking. However, the EMD method generally suffers from mode mixing, and the EEMD algorithm was proposed to resolve this issue [97]. Thus, employment of EEMD in future studies might result in a more accurate denoising of SCG signals during walking, and eventually better estimation of cardiac time intervals such as PEP. Other noise reduction methods, including wavelet transform, adaptive filters, and morphological techniques (e.g., using top hat transform), were also used to remove noise from SCG signals [95].

Daily physical activities, such as walking, introduce noise into the recorded SCG signals and affect their morphology. Therefore, techniques that can remove noise from ambulatory SCG are essential. One study [98] used an evolving fuzzy neural network algorithm to identify the SCG cycles polluted by movement artefacts and remove them from the SCG signal. In another effort [72], a normalized least-mean-square (NLMS) adaptive filter was utilized to cancel the motion noise from SCG of ambulatory subjects. The results of that study showed that adaptive filtering was promising in denoising SCG signals captured during walking. To improve these results, another study [99] utilized a dual-sensor approach where the SCG signals from the anterior and posterior chest wall were acquired. An NLMS adaptive filter algorithm was then used to remove the motion artefact from SCG signal. The noise cancellation performance was calculated and compared for five different reference sensor placement spots around the chest wall. Results showed that using two SCG sensors can lead to a better motion noise cancellation than using a single sensor. Using multiple sensors, however, will increase system complexity.

Some studies pointed out the importance of assessing day-to-day variability when developing a robust system of SCG analysis. Pouyan et al. [100] proposed an algorithm based on a graph-mining technique, called graph similarity score, which was robust to noise and day-to-day variability and could be used to evaluate the risk of HF-related exacerbations for patients at home. A summary of noise removal techniques utilized for SCG denoising is listed in Table 3. More studies are needed that compare different filtering methods in clinical and ambulatory settings.

### 2.2. Segmentation

Signal segmentation is one of the first steps in the processing of SCG signals. Segmentation is the process of finding SCG events (i.e., cardiac cycles) in the SCG signal. Different methods and algorithms were used for SCG segmentation. For example, Jain and Tiwari [64] proposed a three-step algorithm where the signal was first filtered using a denoising algorithm based on discrete wavelet transform. The denoised signal peaks were then detected using an adaptive threshold based on Otsu’s method. The first and second components of SCG (corresponding to the first and second heart sounds, i.e., S1 and S2) were finally identified based on the signal energy. Other methods, such as matched filtering with a template consisting of previously identified SCG events, were also used for SCG segmentation [66,67].

### 2.3. Feature Extraction

Feature extraction is yet another step of SCG signal processing. Identifying the most significant signal features can result in efficient signal classification since these features are eventually the inputs to machine learning algorithms. Determining the most effective and accurate techniques to extract specific signal features is a necessary step that should be done before identification of useful features. For example, there are different methods for estimating the time-frequency distribution of the SCG signal. Every method has its own advantages and disadvantages, and might be suitable for certain types of signals or under certain conditions. Several studies were done to determine the most accurate methods for extracting time-frequency features of the SCG signals [67,104,105]. In these studies, different time-frequency distribution techniques were utilized, including short-time Fourier transform [67,104,105], polynomial chirplet transform (PCT) [67,105], wavelet transform with different mother functions [67], Wigner–Ville distribution, and smoothed pseudo Wigner–Ville distribution (SPWVD) [105]. PCT and SPWVD were found to have the most accurate time–frequency distribution estimations and appeared more suited for determining the frequency content of SCG signals. Using these methods, SCG signals of healthy subjects were found to contain three main spectral peaks below 100 Hz.

Historically, feature extraction of SCG signals mostly focused on the time domain and the frequency domain, separately. The time domain features include statistical features, such as mean, median, and standard deviation, and features related to cardiac mechanics, such as cardiac time intervals. The frequency domain features include statistical features and frequency coefficients obtained from fast Fourier transform (FFT).

#### 2.3.1. Time-Domain Features

Statistical time-domain features include those based on the entire signal, and those from divided segments of the signal. Features from segments of the SCG signal were obtained by dividing the SCG signal into a specific number of equal-sized bins and calculating the arithmetic mean of each bin as a feature [56,106]. Similarly, one study divided the signal into bins; however, binning of the signal was performed discriminately, where the signal portions corresponding to higher variation received a higher concentration of bins [65]. That algorithm divided the bin corresponding the highest standard deviation in a recursive fashion, until some criteria, such as reaching the desired number of bins, was met. Other statistical time-domain features, such as mean, kurtosis, skewness, and standard deviation were also extracted from the SCG signal [41]. Time-domain features also included features related to cardiac mechanics, heart rate and heart rate variability, and turning point ratios [47,48]. In addition, when the ECG R and Q information is concurrently available with the SCG fiducial points (AO, AC, MO, and MC), certain intuitive time-domain features can be determined. These include CTIs (e.g., PEP, isovolumic contraction time (IVCT), LVET, and isovolumic relaxation time (IVRT)) and other metrics such as PEP/LVET ratio, (IVCT+IVRT)/LVET (also called myocardial performance index), and the LVET/R-R-interval ratio [81,107]. Amplitudes and slopes associated with the fiducial points, such as MC to AO slope, were used in some studies [61,107], as well as features of the SCG signal that do not depend on specific fiducial points, such as maxima, minima, and their associated widths of specific segments of the SCG signal [82].

#### 2.3.2. Frequency-Domain Features

Statistical frequency-domain features include those obtained from various frequency bands, and across the entire available frequency spectrum. Features from the frequency bands of an averaged triaxial SCG signal were obtained by calculating the median of each band [78]. One study calculated the approximate and spectral entropy of the 0–11-Hz frequency band [47]. The average power of various frequency bands (0–3 Hz, 3–6 Hz, …, 15–18 Hz) was also utilized [41]. Various statistical metrics, such as arithmetic mean, median, standard deviation, skewness, kurtosis, mode, average power, sample entropy, spectral entropy, and the Kolmogorov complexity, were also calculated across the entire available frequency band [40,41].

Other frequency-domain features include frequency coefficients such as amplitudes and frequencies. Features were either obtained by taking the frequency amplitudes across a range of the frequency spectrum (0–512 Hz) [56], or by taking the frequencies and amplitudes at specific peaks of the spectrum, such as the first, second, and third peaks [41].

In summary, successful feature extraction from SCG signals results in a more efficient classification of these signals. Different studies that investigated the utility of various feature extraction methods/algorithms in both time and frequency domains were described in this section. However, more studies can possibly lead to improve the available methods and define more effective features. In addition, the features currently extracted from SCG signals can be categorized into intuitive (e.g., LVET) and non-intuitive (e.g., skewness) features. Future studies can also address the question of which intuitive or non-intuitive features can be more useful in classification of SCG signals. A summary of these features are listed in Table 4.

### 2.4. Machine Learning

Applications of predictive methods such as machine learning are increasingly being used in biomedical signal processing, including for SCG analysis. Much inter- and intra-subject variability exists in SCG signals and machine learning can be used to automatically recognize the underlying patterns. Some of the applications of machine learning techniques include detection of cardiovascular disease, cardiac mechanics, and parameters affecting SCG waveform such as respiration cycles.

Some studies sought to use classification tools such as support vector machines (SVM) and neural networks (NN) to automatically detect cardiovascular disease. An early study using NNs [107] classified patients based on their SCG as either having coronary artery disease (CAD) or as low risk/normal. They predicted CAD with a sensitivity of 80% and a specificity of 80%. Recent studies [47,48] sought to classify cardiovascular conditions with SCG signals obtained via a smartphone’s inertial measurement unit (IMU). A multi-class classifier was used [48] to classify subjects as either having ST-elevation myocardial infarction, having atrial fibrillation, being preferred for percutaneous coronary intervention procedure, or normal. The proposed classifier achieved classification accuracies between 70 and 79%. However, the same study [48] created a binary classifier (normal vs. atrial fibrillation) and achieved an accuracy of 98.7% using an SVM.

Classification was also used to detect the respiration cycles (inspiration and expiration) [56,106] and lung volume (high and low lung volume) [40,65]. In one study [56] classifying respiration cycles, two different training scenarios were implemented. The first was a leave-one-subject-out (LOSO) approach, which trained the SVM on all but one subject, and tested on the subject who was left out. The second was a subject-specific (SS) approach, which trained and tested on each subject individually. The average accuracies for LOSO and SS were 88.1% and 95.4% respectively. Other studies [40,65] sought to classify SCG signals according to the lung volume phases as opposed to inspiration/expiration.

Classification methods were also utilized to help identify fiducial points on the SCG signals [61], artefact presence in the SCG [98], and identification of the sensor location [41,82].

Other machine learning methods were used on SCG signals such as hidden Markov models (HMM) and graph similarity analysis. An HMM-based method was used in one study [108] to estimate the heart rate, heart rate variability, and CTIs from an SCG signal. A graph similarity analysis [78] was used in another study through the use of k-nearest neighbor graphs on SCG signals from HF patients to identify them as compensated (outpatient) or decompensated (hospitalized).

In summary, machine learning algorithms were used for different purposes in SCG studies, including SCG classification into different phases of respiratory cycle (e.g., high vs. low lung volume), determining fiducial points (e.g., IM and AO) and cardiac time intervals (e.g., PEP), and classification of subjects into patients and low risk/normal. A summary of the machine learning algorithms used for SCG analysis is listed in Table 5.

## 3. Recent Human Studies Suggesting Clinical Utilities

Early use of SCG for cardiac diagnosis faced obstacles such as the large instrumentation size and unclear understanding of the signal characteristics and inter- and intra-subject variabilities. However, recent advances in sensor technologies and signal processing methods led, at least in part, to new numerous studies that provided better insight into these issues. The high morbidity and mortality associated with cardiovascular disease and the high cost of care may have provided motivation to more studies that re-evaluated the feasibility and utility of seismocardiography for diagnosis and monitoring of cardiac function [77,111,112]. Some of the studies reviewed here focused on telemonitoring of cardiac time intervals and heart rates.

### 3.1. Portable Detection of SCG

Wearable technologies can continuously monitor cardiac activity outside clinics and hospitals. This continuous monitoring might help in early detection of serious cardiac conditions, which can enable timely intervention and potentially reduce healthcare costs. Most current wearable cardiac activity monitoring techniques are based on ECG measurements. However, recent studies proposed wearable SCG systems for the assessment of the mechanical aspects of cardiovascular function, including relative changes in cardiac output, contractility, and blood pressure [113]. SCG wearable monitors might be used to assess myocardial contractility via pre-ejection period (PEP) [81]. Another wearable system utilized triaxial accelerometers and gyroscopes to record all six axial and rotational components of the SCG signals [36]. The rotational vibration about the longitudinal (head-to-foot) axis showed a lower sensitivity to walking noise than other components, which might be useful for annotation of SCG signals in ambulant subjects [36].

Today, smartphones and smartwatches are common and can be used as part of telemedicine for real-time patient monitoring at a relatively low cost. Smartphones used SCG for continuous monitoring of heart rate variability [114], and cardiac activity of patients suffering from heart disease [115]. In a recent study, the feasibility and accuracy of measuring heart rate using a smartphone accelerometer was assessed in different postural positions [46] and suggested utility of SCG for heart rate estimation.

Wearable SCGs might be contaminated with different type of noise. Therefore, investigating the effective noise removal techniques for ambulatory subjects is needed. A few ongoing studies are addressing this question. These studies were described in Section 2.1.

### 3.2. Heart Rate Monitoring

Heart rate (HR) monitoring is a common way to monitor cardiovascular function, and can identify some abnormalities such as arrhythmia. Traditional HR estimation methods are mostly based on ECG signal processing. SCG signals can also be used for HR estimation. SCG-based HR estimation algorithms are not usually developed to replace the current HR monitoring methods. Instead, SCG-based HR estimations can be used as a feature in other studies that focus on the clinical utility of SCG, since recent studies revealed that SCG can reliably detect HR in the absence of other modalities such as ECG. For example, Cosoli et al. [26] suggested a general algorithm that can estimate HR from various signals, including SCG, ECG, phonocardiogram (PCG), and PPG. Considering the ECG signal as a gold standard, the SCG HR estimation was more accurate than the estimations from the PCG and PPG signals. Wahlstrom et al. [108] used an HMM to determine different stages of a cardiac cycle, which were used for estimating beat-to-beat intervals. The beat-to-beat intervals of the SCG signal were then utilized for HR and HR variability estimations. Mafi [116] suggested an algorithm based on empirical mode decomposition and empirical wavelet transform that can extract HR from SCG signals. Tadi et al. [25] used a Hilbert adaptive beat identification technique to determine the heartbeat timings and inter-beat time intervals from SCG signals. An android application was implemented based on this algorithm that could monitor the subject heart rate in real time using a smartphone accelerometer. Tadi et al. [69] proposed an algorithm based on S-transform, Shannon energy, and successive mean quantization transform to identify heartbeat and beat-to-beat interval from SCGs. The algorithms proposed in the latter two studies had a high agreement with the ECG HR. Taebi et al. [39] used SCG signals in the dorso-ventral direction to estimate the HR during different phases of respiration in real time. Their results showed that normal subjects have a different HR during high and low lung volumes. In a recent study [46], the heart rate was derived from a smartphone SCG signal, and compared to that extracted from ECG. Results showed that the HR provided by SCG, particularly in the dorso-ventral direction of the supine position, was equivalent to that provided by conventional ECG.

### 3.3. Pulse Transit Time

SCG is used to estimate different cardiovascular parameters such as cardiac time intervals, pulse transit time, and blood pressure. For example, non-contact SCG was used at different body locations for estimating central arterial pressure and carotid arterial pressure waveforms [49,117,118]. Pulse transit time might be estimated from the time difference between AO point on the xiphoid SCG and AO point on the carotid SCG [119]. Blood pressure changes can be monitored using pulse transit time. For this purpose, the pulse transit time, which was defined as the time required for the blood pressure wave to travel from one location to another [120], was first measured from the SCG signals [121]. The measured pulse transit time was then used to estimate the patient blood pressure [79]. Based on similar techniques, a wrist-watch, consisting of an accelerometer and an optical sensor, was developed to monitor blood pressure [122]. In this “SeismoWatch”, the blood pressure was estimated from the travel time of the micro-vibrations propagating from the heart to the wrist when the watch was held against the subject’s sternum. In a different study, Di Rienzo et al. [73] developed a system that measures SCG and PPG at multiple locations alongside the ECG signal. The pulse transit time may then be derived from the PPG.

### 3.4. Cardiac Time Intervals

Cardiac time intervals were used for a long time for cardiovascular disease diagnosis [123,124]. There are various SCG-based algorithms with different levels of accuracy that were proposed for estimating cardiac time intervals such as PEP, LVET, ICT, systolic time, and diastolic time in healthy subjects and patients with previous heart conditions [61,108]. For example, LVET might be estimated from SCG signals that are acquired using LDV and microwave Doppler radar [51,117]. The LVET value from non-contact SCG was similar to the value derived from a photoplethysmogram (PPG) [117]. Rivero et al. [125] proposed a new algorithm that uses continuous wavelet transform as a base to determine the aortic valve opening and isovolumic moment points on the SCG signal. The electro-mechanical window (EMw) is defined as the duration between the electrical and mechanical systole. EMw is a potential biomarker that can be utilized for diagnosing several cardiovascular diseases. ECG and PCG signals are conventionally used to determine EMw. However, Jain et al. [57] showed that SCG is a suitable alternative to PCG for estimating the EMw.

Analysis of SCG data recorded from the sleep patterns of a subject aboard the International Space Station (in microgravity) resulted in accurate identification of cardiac time intervals and SCG fiducial points (such as AO, AC, MO, MC, LVET, and PEP) with implications for future clinical application [81]. As described earlier, SCG morphology is affected by different factors such as the sensor location and respiration. Investigating the effect of these factors on the estimation of cardiac time intervals from SCG signals can possibly reveal clinically useful information.

### 3.5. SCG in Patients with Cardiac Conditions

In addition to human studies on healthy populations, there were several studies that focused on the application of SCG in patients with cardiovascular disease. SCG signals were used for diagnosis and monitoring of different clinical conditions such as atrial fibrillation [47,48,68,70], atrial flutter [55], heart valve disease [37,44,80], coronary artery disease and ischemia [9,10,48], myocardial infarction [126], heart failure [75,78,82,100,119], structural heart disease [80], and heart stress testing [58].

According to a 2017 report, the prevalence of any heart valve disease is 2.5% of the United States population [127]. Heart sounds that are believed to be generated by opening and closure of heart valves can be used as a diagnostic marker of these diseases. Stethoscope and PCG are the common conventional methods for heart sound monitoring. SCG signals were reported as a potential efficient alternative for PCG signals for monitoring of heart sound signals [44].

ECG is currently the main diagnostic method of atrial fibrillation (AF). A preclinical study [68] investigated the usefulness of SCG for AF detection. Results suggested that the amplitude of the SCG signal correlates to beat interval and significantly varies from beat to beat during AF. This study also suggested that the combination of SCG and ECG may reveal certain behavior in the electromechanical delay characteristic of AF, which may lead to extra indicators for early detection of AF.

Paukkunen et al. [55] showed that three-dimensional (3D) vector trajectory of SCG might be useful in diagnosing atrial flutter. The results of this study suggested that the intra-subject correlation of 3D SCGs was strong. However, the signals had a very weak inter-subject correlation. Future studies might prove the utility of SCG 3D vector trajectory for diagnosis of different cardiovascular disease and abnormalities.

## 4. Conclusions, Limitations, and Future Directions

Growth in the field of seismocardiography accelerated during the last decade. However, open issues and limitations hamper its clinical application. Reviewed here are some of the current limitations along with potential future work.

- SCG variability: SCG morphology is affected by different factors such as respiration, sensor location, subject posture, the amount of chest surface soft tissue, and different heart diseases. Although studies investigated some of these factors, further research is needed to adequately account for SCG variations. The results of such investigations might improve utility for cardiac disease diagnosis and monitoring.
- Lack of accepted standard for the cardio-mechanical SCG fiducial points: A small number of recent studies focused on robust documentation of the relationship between fiducial points and their physiological sources. In addition, SCG morphology changes with different factors (e.g., sensor location, patient posture, etc.). It would be useful to investigate the effect of these factors on the SCG signal fiducial points.
- SCG genesis: Although several studies aimed to elucidate the physiological source(s) of the SCG signals, much work remains to be done. SCG signals are likely affected by extra-cardiac factors including respiration and intrathoracic pressure. Therefore, considering these parameters may further help elucidate SCG sources.
- Computational models: Realistic computational simulations utilizing finite element and other modeling methodologies with realistic geometries and material properties might be helpful in predicting the effects of varied cardiac conditions on SCG features.
- Library of SCG signals: A common comprehensive database would provide a basis for researchers interested in analyzing SCG and other biomedical signals. The MIT-BIH arrhythmia database is a good example of a biomedical signals database. This database plays an important role in stimulating both basic research and medical device development. A similar SCG database would attract more researchers to investigate and compare the performance of different algorithms and approaches.
- Correlation between SCG and other electro-mechanical signals: Combining information from ECG, PCG, and BCG with that of SCG may lead to a hybrid modality with increased diagnostic utility of cardiac disease. This may result in more complex features that require increase use of machine learning approaches.

In conclusion, signal processing techniques and physiologic understandings rigorously applied may transform SCG signal analysis from a research interest to a powerful bedside or home monitoring tool.

## Figures and Tables

**Table 1.** Seismocardiography (SCG) feature points pointed out in the literature.

Feature Point	Reference
Peak of atrial systole (AS)	[10,14,30]
Mitral valve closure (MC)	[10,14,28,30,31]
Peak of rapid systolic ejection (RE)	[10,14,30,32]
Peak of rapid diastolic filling (RF)	[10,14,30]
Isovolumic contraction (IC)	[10]
Mitral valve opening (MO)	[14,28,30,31]
Aortic valve closure (AC)	[14,28,30–32]
Aortic valve opening (AO)	[14,28,30–33]
Isovolumic movement (IM)	[14]
Rapid diastolic filling time	[14]
Isotonic contraction (IC)	[14]
Isovolumic relaxation time (IVRT)	[14,28,31]
Left ventricular ejection time (LVET)	[14,28,31,32]
Maximum acceleration in aorta (MA)	[28,32]
Pre-ejection period (PEP)	[28,31,32]
Total electromechanical systole period (QS2)	[28,31,32]
Maximum blood injection (MI)	[28]
Isovolumic contraction time (IVCT)	[28,31]
Left ventricular lateral wall contraction peak velocity (LCV)	[34]
Septal wall contraction peak velocity (SCV)	[34]
Trans-aortic peak flow (AF)	[34]
Trans-pulmonary peak flow (PF)	[34]
Trans-mitral ventricular relaxation flow (MF_E_)	[34]
Atrial contraction flow (MF_A_)	[34]

**Table 2.** Summary of acceleration sensors used for SCG data acquisition. Abbreviations used in the table: Acc—accelerometer; Gyr—gyroscope; ARS—angular rate sensor; 1—uniaxial; 2—biaxial; 3—triaxial; MEMS—micro electromechanical systems; SP—smart phone.

Reference	Sensor Type	Sensor Model	Sensor Location
[54–56]	3-Acc	SCA610-C21H1A, Murata Electronic	1 cm above xiphoid
[43,44]	3-MEMS-Acc	MMA 7361, Freescale Semiconductor	Heart apex
[57]	3-MEMS-Acc	MMA 7361, Freescale Semiconductor	Above xiphoid
[58]	3-MEMS-Acc	Analog Devices	2 cm above xiphoid
[36,38,45]	3-MEMS-Acc 3-MEMS-Gyr	KXRB5-2042, Kionix MPU9150, Invensense	Left sternal border along the 3rd rib
[59]	3-Acc	ViSi Mobile, Sotera Wireless	Chest wall
[60,61]	1-Acc 1-Acc	4381, Brüel & Kjær 393C, PCB Piezotronics	Above xiphoid
[62,63]	1-Acc	DS1104, DSPACE	Xiphoid process
[64]	3-Acc	ADXL 335, Analog Devices	Chest wall
[46]	3-SP-Acc	iPhone6, Apple	Midclavicular line and 4th intercostal space Belly above navel
[65,66]	3-Acc	356A32, PCB Piezotronics	Left sternal border along the 4th intercostal space
[67]	3-Acc	X6-2mini, GCDC	Left sternal border along the 4th intercostal space
[68]	1-MEMS-Acc	SCA620, Murata Electronic	Sternum—anterior chest
[25,69,70]	3-MEMS-Acc	MMA8451Q, Freescale Semiconductor	Sternum
[34,71]	1-Acc	LIS331DLH, STMicroelectronics	Mitral valve, tricuspid valve, aortic valve, pulmonary valve
[72]	3-MEMS-Acc	MMA 7361, Freescale Semiconductor	Left sternal border along the 3rd rib
[73]	3-MEMS-Acc	MMA8451Q, Freescale Semiconductor	Sternum, aortic valve, heart apex
[74]	3-Acc 1-Acc	CXL01LF3, Crossbow Technology 7290-A, Endevco Microtron	Manubrium Xiphoid
[75–78]	3-Acc	BMA280, Bosch Sensortec GmbH	Mid-sternum
[79]	3-MEMS-Acc	TSD109C, Biopac Systems	Left sternal border along the 3rd rib
[41]	3-Acc	356A32, PCB Piezotronics	Sternum, upper and lower sternum
[80]	1-Acc	N/A	Sternum
[81]	3-MEMS-Acc 3-Gyr	MMA8451Q, Freescale Semiconductor L3GD20, STMicroelectronics	N/A
[82]	3-Acc	ADXL 335, Analog Devices	Mid-sternum, upper sternum, lower sternum Point of max impulse, below left clavicle, below right clavicle
[83]	3-MEMS-Acc 3-MEMS-Gyr	SparkFun, Intel Edison	Sensor clipped on subjects clothes
[50,51]	Microwave Doppler radar		
[47,48]	3-SP-Acc	Xperia Z-series, Sony	Chest
[49]	Laser Doppler vibrometer	PDV-100, Polytec	
[37]	3-MEMS-Acc 2-MEMS-Gyr	LIS344ALH, STMicroelectronics LPY403AL, STMicroelectronics	Heart apex Lower back of subject between 2nd and 3rd lumbar vertebrae
[31]	3-MEMS-Acc 3-MEMS-ARS	MMA8451Q, Freescale Semiconductor MAX21000, Maxim Integrated	Sternum
[53]	AUSMC	Composed of the following sensors: - MA40S4S, Murata Electronics - FG-23629 Knowles microphone	∼30 × 40 cm^2^ thoracic and abdominal surface

**Table 3.** Summary of the noise removal methods used for SCG filtration.

Method	Application	Reference
low-, band-, high-pass, notch filtering	Baseline wandering, breathing and body movement artefact removal	[26,36,38,41,45,46,55,58–63,67,71,75,76,78–80,82,93]
Adaptive filtering	Motion artefact removal	[88,95]
Averaging theory	Motion artefact removal	[101]
Comb filtering	Removing respiration noise from radar signal	[50]
Empirical mode decomposition	Baseline wandering, breathing and body movement artefact removal	[76,94,95]
Independent component analysis	Motion artefact removal	[102]
Median filtering		[96]
Morphological filtering		[95]
Polynomial smoothing	Motion artefact removal	[103]
Savitzky–Golay filtering	Motion artefact removal	[83,103]
Wavelet denoising	Segmentation of HSs and SCG	[64,95,96]
Wiener filtering		[94]

**Table 4.** Summary of the features used in machine learning algorithms for SCG signal analysis.

SCG Features	Reference
Simple time domain	[47,61,81,82,107]
Statistical time domain	[41,56,65,106]
Simple frequency domain	[41,56]
Statistical frequency domain	[40,41,47,78]

**Table 5.** Summary of the machine learning algorithms used for SCG signal analysis. NN—neural network; SVM—support vector machine; HMM—hidden Markov model; *k*-NN—*k*-nearest neighbors; EFuNN—Evolving Fuzzy Neural Network.

		Reference
Classification	NN	[107,109]
	EFuNN	[98,110]
	SVM	[40,47,56,65,106,109]
	Random forest	[47,109]
	Logistic regression	[61]
	J48 decision tree	[41]

Clustering	*k*-means	[109]

Regression	Xgboost	[82]

Graph-Similarity	*k*-NN graph	[78]

HMM	Viterbi sequence	[108]
